# Fusion of ConvNeXt-Tiny and Swin-Tiny backbones: a comparative analysis for diabetic retinopathy classification

**DOI:** 10.3389/frai.2026.1812599

**Published:** 2026-07-03

**Authors:** J. Paranthaman, Sathya Pichandi, Aparna Mohanty

**Affiliations:** School of Electronics Engineering, Vellore Institute of Technology, Vellore, India

**Keywords:** APTOS 2019 dataset, channel-spatial attention module (CSAM), Contrast Limited Adaptive Histogram Equalization (CLAHE), ConvNeXt-Tiny, diabetic retinopathy, EyePACS Combined dataset, ImageNet-1k dataset, Swin-Tiny transformer

## Abstract

Diabetic retinopathy (DR) is a leading cause of preventable blindness, which has motivated the development of reliable automated grading systems on retinal fundus images. In this study, we perform a controlled comparative evaluation of ConvNeXt-Tiny, Swin-Tiny and their feature fusion for DR classification using the Asia Pacific Tele-Ophthalmology Society (APTOS) 2019 dataset. All models were initialized with weights pre-trained on ImageNet-1K and evaluated with two transfer learning strategies: direct fine-tuning on APTOS 2019, and EyePACS-based domain adaptation with task-specific fine-tuning. Systematic ablation experiments were carried out to evaluate the contribution of Contrast Limited Adaptive Histogram Equalization (CLAHE) preprocessing and channel-spatial attention modules (CSAM). We carried out experiments on the APTOS 2019 dataset with fixed train, validation and test splits and evaluated model stability across three runs with different random seeds by reporting mean ± standard deviation of performance metrics, while performance varied widely across architectures and training settings. After domain adaptation, the fusion-based models achieved more balanced results, while the standalone Swin-Tiny showed weaker adaptation to the retinal imaging domain, and was less sensitive to subtle lesion patterns under the EyePACS-based transfer learning. Adding CLAHE preprocessing and CSAM integration did not consistently improve class-balanced metrics. The best fusion configuration achieved a mean test accuracy of 88.34% ± 1.09 and a macro F1-score of 0.7376 ± 0.0183 on the APTOS 2019 dataset across repeated runs. These results suggest that domain-specific adaptation and architectural complementarity are more beneficial in boosting DR classification performance than auxiliary preprocessing or attention enhancement. The study also emphasizes the importance of controlled comparative evaluation, stability analysis, and configuration-specific evaluation in the research of medical image classification.

## 1 Introduction

Diabetic retinopathy (DR) is one of the leading causes of preventable blindness worldwide and remains a major complication of diabetes mellitus (Zhao et al., 2024; Ma et al., 2023). Early detection and accurate severity grading are essential for timely clinical intervention, yet manual screening of retinal fundus images is labor intensive and subject to inter-observer variability. Automated deep learning based DR grading systems therefore continue to attract significant research interest for supporting large scale screening and clinical decision making. Despite substantial progress, DR classification remains challenging because retinal lesions such as microaneurysms, hemorrhages, and exudates are often small, visually subtle, and unevenly distributed across retinal regions (Zhao et al., 2024; Ma et al., 2023; He et al., 2016). Variability in illumination, imaging quality, class imbalance, and inter-stage similarity further complicate automated grading. Recent convolutional neural network (CNN) based approaches have demonstrated strong capability in extracting localized pathological features from fundus images and have achieved promising DR classification performance (He et al., 2016; Zhou et al., 2018). Several recent CNN-based diabetic retinopathy classification studies have continued to demonstrate strong performance using optimized deep learning and transfer learning strategies. For example, Sadek et al. (2025) reported effective DR grading performance across multiple retinal imaging datasets using CNN-based architectures and preprocessing techniques, further supporting the continued relevance of convolutional representations for retinal lesion analysis and automated DR grading.

More recently, transformer based vision architectures have been explored for retinal image analysis because of their ability to model long range spatial relationships through self attention mechanisms (Dosovitskiy, 2021; Li et al., 2022). Hierarchical transformer variants such as Swin Transformer reduce computational complexity while preserving multi scale feature representation. However, transformer based models in medical imaging may remain sensitive to dataset scale, class imbalance, and domain variability, particularly when lesion level discrimination is required. In retinal fundus analysis, the relative advantages of CNN based and transformer based representations therefore remain dependent on training configuration and dataset characteristics.

Among lightweight modern architectures, ConvNeXt-Tiny and Swin-Tiny provide an informative comparison because they represent two distinct but computationally efficient design paradigms. ConvNeXt-Tiny modernizes CNN design while preserving convolutional inductive biases that are beneficial for localized feature extraction. Swin-Tiny introduces hierarchical window based self attention for broader contextual representation with manageable computational cost. Prior studies have suggested that hybrid CNN–transformer frameworks may combine complementary representational characteristics, although the specific contribution of architectural fusion in DR grading remains inconsistently reported across datasets and evaluation protocols. Recent hybrid medical imaging studies have also explored combining transformer-based global representation learning with graph- or convolution-based local feature modeling to improve diagnostic performance under limited data conditions (Xu et al., 2025). These findings further support the motivation for investigating complementary feature fusion strategies in medical image classification tasks. In addition to hybrid representation learning, recent studies have also investigated robustness challenges associated with limited or imperfectly labeled medical datasets. For example, Wu et al. (2026) discussed the implications of informative missingness in semi-supervised learning and emphasized the importance of robust learning strategies under incomplete data conditions. Although the present study focuses on supervised diabetic retinopathy classification, these broader developments further highlight the importance of carefully controlled evaluation frameworks and robustness analysis in medical image learning systems. Another important challenge concerns transfer learning and domain adaptation. Most retinal image classification studies initialize models using ImageNet-1K pretrained weights, even though natural image statistics differ substantially from retinal fundus imagery. Domain specific adaptation using large retinal datasets such as EyePACS may improve feature alignment, yet its effect across CNN, transformer, and fusion architectures is not fully characterized. Similarly, commonly used enhancement and attention techniques, including Contrast Limited Adaptive Histogram Equalization (CLAHE) and channel–spatial attention modules (CSAM), are frequently incorporated without systematic evaluation of their independent contribution.

Motivated by these gaps, this study presents a controlled comparative evaluation of ConvNeXt-Tiny, Swin-Tiny, and their feature level fusion for diabetic retinopathy classification within a unified experimental framework. Two transfer learning strategies were investigated: direct fine tuning from ImageNet-1K initialization and domain specific adaptation using the EyePACS combined dataset followed by task specific fine tuning on Asia Pacific Tele-Ophthalmology Society (APTOS) 2019. In addition, systematic ablation experiments were conducted to evaluate the contribution of CLAHE preprocessing and CSAM integration. The study emphasizes configuration dependent behavior, architecture specific adaptation effects, and balanced reporting of both positive and negative findings to support more reproducible and practically grounded DR classification research.

**Figure 1:**
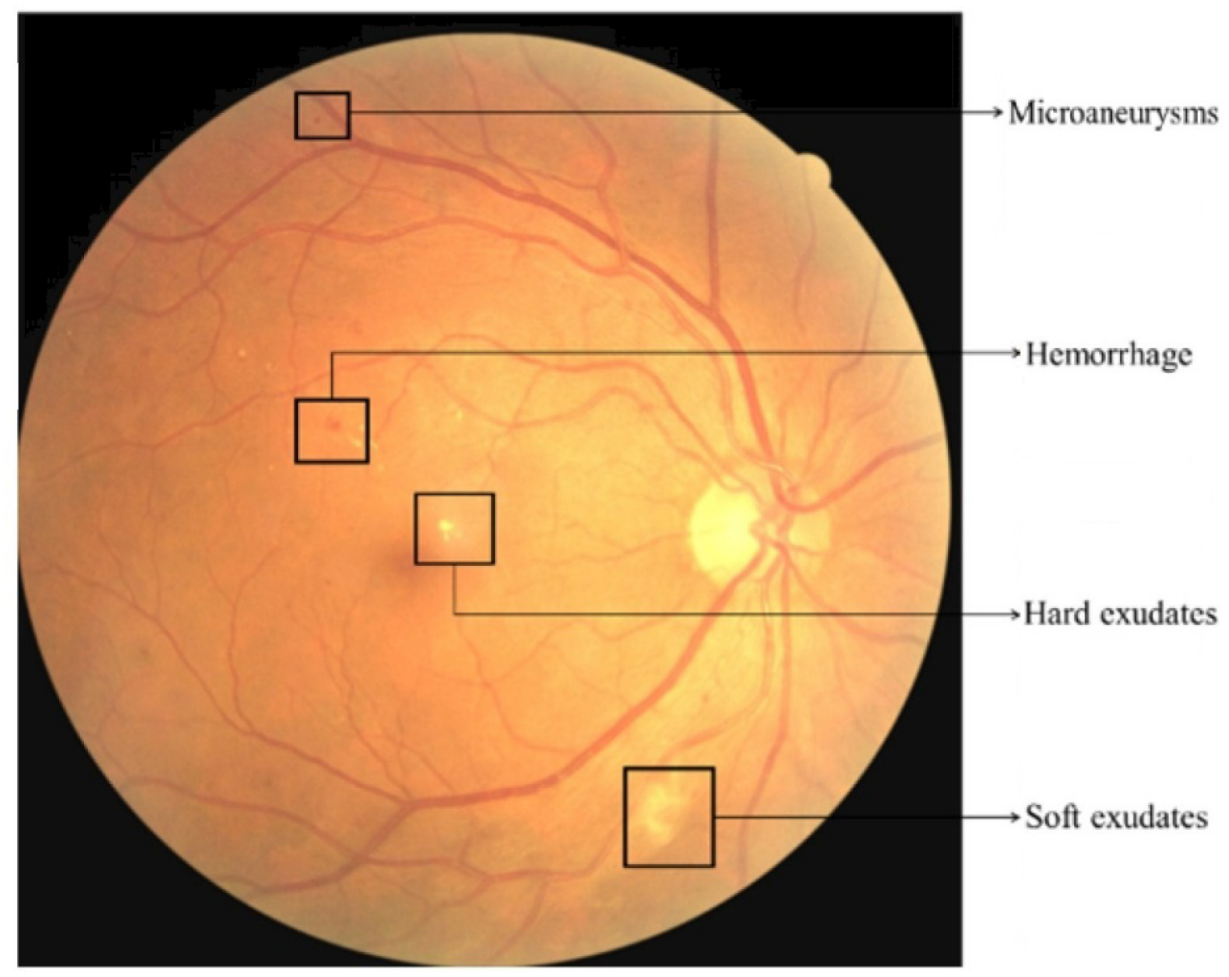
Illustrative fundus image with diabetic retinopathy. Adapted from Alyoubi et al. (2021), licensed under CC BY 4.0.

Diabetic retinopathy manifests through retinal abnormalities such as microaneurysms, hemorrhages, and exudates that are directly observable in fundus images and form the basis of automated grading systems. Figure 1 illustrates representative retinal lesions, while Table 1 summarizes the DR severity grading stages considered in this study. Unlike prior hybrid DR classification studies that primarily focus on proposing increasingly complex architectures, this work emphasizes a controlled comparative evaluation of CNN-based, transformer-based, and hybrid fusion paradigms under unified experimental conditions. The primary contribution of this study is a systematic comparative analysis of architecture-dependent behavior, domain adaptation effects, and the practical impact of preprocessing and attention mechanisms on class-balanced diabetic retinopathy classification performance.

**Figure 2:**
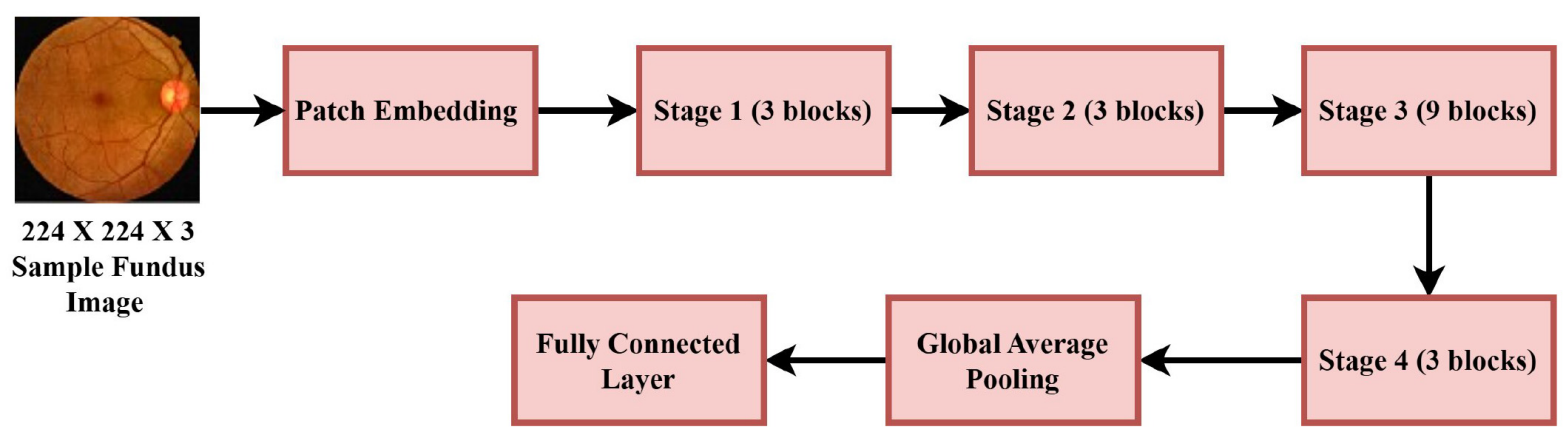
The ConvNeXt-Tiny architecture (Liu et al., 2022).

**Figure 3:**
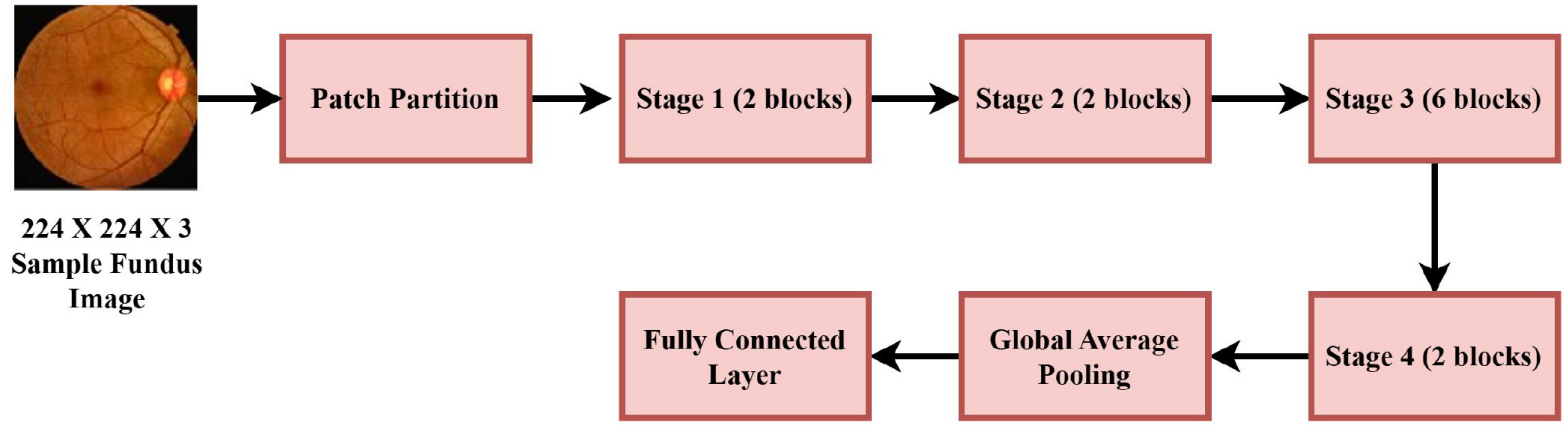
The Swin-Tiny architecture (Liu et al., 2021).

**Table 1:** Stages of diabetic retinopathy and their clinical manifestations (Das et al., 2021).

Diabetic retinopathy level of severity	Observations noted during dilated ophthalmoscopy
No diabetic retinopathy	No observable retinal abnormalities or lesions are present.
Mild non-proliferative diabetic retinopathy (NPDR)	Characterized by the presence of isolated microaneurysms without additional retinal abnormalities.
Moderate NPDR	Marked by an increased number of microaneurysms and intraretinal hemorrhages, which may be accompanied by features such as cotton-wool spots or hard exudates.
Severe NPDR	Defined according to the 4:2:1 rule, involving extensive intraretinal hemorrhages and microaneurysms across four quadrants, venous beading in at least two quadrants, or prominent intraretinal microvascular abnormalities in one quadrant, with no evidence of neovascularization.
Proliferative diabetic retinopathy (PDR)	Characterized by the formation of pathological new blood vessels on the retinal surface or optic disc, which may result in vitreous or preretinal hemorrhage and pose a high risk of severe visual impairment.

### 1.1 Highlights of this work

- A controlled comparative benchmark across CNN based, Transformer based, and hybrid fusion architectures using a unified training protocol has been conducted.
- A systematic analysis was conducted to identify the architecture dependent effects of domain specific adaptation using the EyePACS Combined dataset.
- Structured ablation experiments were performed to measure the impact of preprocessing (CLAHE) and attention mechanisms (CSAM) on class balanced performance metrics.
- A robust configuration has been identified based on ConvNeXt-Tiny & Swin-Tiny feature fusion with domain adaptation and without auxiliary preprocessing or attention modules.

## 2 Related work

### 2.1 CNN-based architectures

Early studies on retinal disease classification predominantly relied on convolutional neural networks due to their strong feature extraction capability. A patch-based CNN was used to localize red lesions in diabetic retinopathy, with strong sensitivity and interpretability supported by lesion-level heat maps (Zago et al., 2020; Prawira et al., 2021; Gour and Khanna, 2021). Comparative retinal disease classification studies showed that AlexNet outperformed VGG16 in multi-label classification settings (Prawira et al., 2021). A two-input VGG16 framework was applied on the Ocular Disease Intelligent Recognition (ODIR) dataset to detect multiple eye diseases simultaneously (Gour and Khanna, 2021). Transfer learning with VGG16 has also been used for DR severity grading, with the introduction of a dedicated ungradable class to improve robustness across DDR, EyePACS Combined, and Indian Diabetic Retinopathy Image Dataset (IDRiD) datasets (Rocha et al., 2022). Approaches focusing on improved computational efficiency and lesion localization have further employed modified CNN architectures (Li et al., 2022). A triple-cascade hierarchical framework, Triple-DRNet, was later proposed for progressive DR stage classification and achieved 92.08% accuracy on the APTOS dataset (Jian et al., 2023). More recently, ConvNeXt repositioned convolutional networks for modern vision tasks using Transformer-inspired design principles. ConvNeXt-Tiny demonstrated competitive performance with Vision Transformers on ImageNet and dense prediction benchmarks (Liu et al., 2022). Subsequent adaptations of ConvNeXt have been reported for malaria detection (Mmileng et al., 2025), explainable monkeypox diagnosis (Waqar et al., 2025), and lightweight quantitative medical applications (Xia et al., 2025). Collectively, these studies confirm the effectiveness of CNNs for lesion detection and grading while also highlighting limitations in global context modeling and multi-label dependency learning.

### 2.2 Transformer-based approaches

Transformers were originally developed for natural language processing and have more recently been adopted in medical imaging due to their ability to model long-range dependencies (Ma et al., 2023; Li et al., 2022; Hou et al., 2022). The Vision Transformer (ViT) demonstrated that pure Transformer models trained on sufficiently large datasets can achieve performance comparable to CNNs (Dosovitskiy, 2021). The Swin Transformer further improved performance for multi-scale detection and segmentation tasks through hierarchical shifted-window attention (Liu et al., 2021). Several transformer models have since been tailored for retinal imaging tasks. A Cross-Field Transformer framework was introduced for DR grading (Hou et al., 2022). Comparative studies evaluating ViT, Swin-Tiny, and ConvNeXt-Tiny for glaucoma detection reported superior performance from transformer-based models (Mallick et al., 2022). Swin-Tiny–based architectures have also been integrated into ophthalmic frameworks, including multi-branch DR grading networks (Liu et al., 2025) and lesion-map with cross-attention fusion strategies for referable DR classification (Mok et al., 2024), along with additional multi-scale fusion approaches. Overall, these works demonstrate strong global feature modeling capability in transformers, although their reduced sensitivity to fine-grained lesion localization has motivated subsequent hybrid approaches (Zhang et al., 2025).

### 2.3 Hybrid CNN–transformer approaches

To overcome the individual limitations of CNNs and Transformers, hybrid models have been developed to combine local and global feature representations. Attention-based feature weighting networks such as AUBNet were introduced to enhance discriminative learning (He et al., 2020). Graph convolutional models with self-supervised learning have been explored for disease correlation modeling (Lin et al., 2021). Multi-label fundus classification frameworks combining graph convolutional modeling with LightGBM have also been proposed (Sun et al., 2022). CNN–Transformer hybrids with dedicated fusion modules have been developed to strengthen cross-representation learning (Hu, 2022). Recent DR studies highlight the effectiveness of ConvNeXt-Tiny and Swin-Tiny hybrid networks for efficient classification (Qezelbash-Chamak and Hicklin, 2025; Madhavi et al., 2025; Khokhar et al., 2025). Cross-feature lesion map fusion methods (Mok et al., 2024), dynamic multi-scale fusion networks (Wang et al., 2024), and prior-guided attention fusion approaches (Xu et al., 2024) have further improved lesion sensitivity in site-specific cases. A KAN-augmented ConvNeXt-Tiny model incorporating Kolmogorov–Arnold Networks was later proposed to enhance classification interpretability (Addya et al., 2025). Comparative evaluations indicate that CNN–Transformer hybrids can effectively integrate lesion localization with global context and may outperform standalone CNN or Transformer models (Zhang et al., 2025).

## 3 Proposed methodology

This study adopts a controlled comparative experimental framework to evaluate convolutional and transformer-based architectures, and their feature fusion, for robust diabetic retinopathy classification. Rather than introducing a new architecture, the objective is to systematically analyze the behavior of complementary deep learning paradigms convolutional neural networks (CNNs) and vision transformers under unified training and evaluation conditions.

Two backbone families are considered: ConvNeXt-Tiny (CNN-based) and Swin-Tiny (transformer-based). In addition to standalone models, a feature fusion configuration combining both backbones is evaluated. Two transfer learning strategies are examined: (i) direct fine-tuning from ImageNet-1K pretrained weights on APTOS 2019, and (ii) domain-specific adaptation using the EyePACS combined dataset prior to final task-specific fine-tuning on APTOS 2019. The motivation for selecting these backbones is based on complementary representational characteristics. ConvNeXt-Tiny provides strong localized feature extraction suitable for detecting fine retinal lesions such as microaneurysms and hemorrhages, while Swin-Tiny offers hierarchical self attention mechanisms for modeling broader contextual relationships. Their fusion enables evaluation of whether combining local and global representations improves class-balanced performance within a unified experimental framework.

In addition, structured ablation experiments are conducted to examine the effects of preprocessing using Contrast Limited Adaptive Histogram Equalization (CLAHE) and feature recalibration using a Channel–Spatial Attention Module (CSAM). These components are evaluated as optional modules across configurations to determine their actual contribution under consistent training settings.

### 3.1 Training configuration

All experiments were conducted on the Kaggle cloud platform using NVIDIA Tesla T4 × 2 GPUs. Fixed train, validation, and test folder splits from the processed APTOS 2019 dataset were used consistently across all experimental configurations to maintain controlled evaluation conditions. Images were resized to 224 × 224 pixels during model training and inference.

For CLAHE-based experiments, preprocessing was performed offline prior to training. Retinal fundus images were converted from RGB to LAB color space, and CLAHE was applied on the luminance channel using a clip limit of 2.0 and a tile grid size of 8 × 8. The enhanced luminance channel was then merged with the original chromatic channels and converted back to RGB space. Subsequently, center cropping and resizing to 512 × 512 pixels were performed before dataset preparation. Training augmentation included random resized cropping, horizontal and vertical flipping, random rotation, random perspective transformation, color jittering, Gaussian blurring, sharpness adjustment, and random erasing. Pixel intensities were normalized using mean and standard deviation values of 0.5 for all RGB channels. All models were optimized using the Adam optimizer with an initial learning rate of 1 × 10^−4^. A cosine annealing learning-rate scheduler was applied during training. Cross-entropy loss with label smoothing (0.1) was used as the optimization objective. Mixed-precision training using automatic mixed precision (AMP) was employed to improve computational efficiency.

A batch size of 32 was used across all experiments. Maximum training duration was controlled through validation-based monitoring and early stopping to reduce overfitting risk and maintain stable optimization behavior across CNN, transformer, and fusion configurations. Preliminary experiments with longer retinal-domain adaptation stages did not consistently improve downstream validation performance and occasionally reduced generalization stability; therefore, the intermediate retinal-domain fine-tuning stage was limited to 10 epochs. Class imbalance was addressed primarily through class-balanced evaluation metrics including macro recall and macro F1-score rather than aggressive resampling or synthetic balancing strategies, in order to preserve the original distribution characteristics of the dataset. Training reproducibility was further supported through deterministic seed initialization across Python, NumPy, PyTorch, and dataloader workers.

### 3.2 Datasets descriptions

#### 3.2.1 APTOS 2019-dataset

The APTOS 2019 Blindness Detection dataset (Asia Pacific Tele-Ophthalmology Society, 2019) was released by the APTOS as part of a Kaggle competition. It contains 3,662 retinal fundus images annotated across the same five Diabetic retinopathy severity grades used in the EyePACS Combined dataset. The class distribution consists of 1,805 images labeled as no DR, 370 as mild DR, 999 as moderate DR, 193 as severe DR, and 294 as proliferative DR. The images in APTOS 2019 are generally more consistent in resolution and visual quality than those in the EyePACS Combined collection, since they were curated by licensed ophthalmologists. In this study, this dataset served as a suitable benchmark for classification evaluation, partly due to its more balanced grade distribution, which helps reduce the effect of class imbalance often present in larger real-world retinal datasets.

#### 3.2.2 The EyePACS Combined dataset

For this study, a publicly available Kaggle repository that aggregates retinal fundus images from multiple diabetic retinopathy benchmarks, including EyePACS, APTOS, and Messidor (Ascanipek, 2024), was used. From this repository, only the dr_unified_v2 subset was selected to maintain label reliability and annotation consistency. This subset contains 92,501 color retinal fundus images labeled according to the International Clinical Diabetic Retinopathy (ICDR) grading scale. Each image is assigned to one of five categories: Grade 0 (no DR), Grade 1 (mild), Grade 2 (moderate), Grade 3 (severe), and Grade 4 (proliferative DR). The dataset includes substantial variation in acquisition devices, imaging conditions, resolutions, and patient populations, which makes it useful for developing and testing robust DR classification models.

### 3.3 Preprocessing strategy (CLAHE) ablation

All input images are first resized and normalized to maintain consistent input dimensions and intensity scaling across models. To study the role of local contrast enhancement, Contrast Limited Adaptive Histogram Equalization (CLAHE) was optionally applied as a preprocessing step. CLAHE can improve the visibility of subtle retinal structures by enhancing local contrast, but it may also increase noise and introduce artifacts. For this reason, CLAHE-enabled and non-CLAHE pipelines are evaluated in parallel within the ablation framework. Conclusions are drawn based on comparative metric outcomes rather than any assumed advantage of contrast enhancement.

### 3.4 Transfer learning and domain adaptation strategy

All backbone networks were initialized with publicly available ImageNet-1K pretrained weights. Two training strategies were evaluated within a unified experimental framework. The first involved direct fine-tuning from ImageNet-1K initialization on APTOS 2019. The second involved domain-specific adaptation using the EyePACS Combined dataset followed by task-specific fine-tuning on APTOS 2019. Domain-specific adaptation was performed on the EyePACS Combined dataset to better align intermediate representations with retinal image characteristics such as vessels, exudates, and hemorrhagic patterns. This stage was treated strictly as domain alignment rather than task pretraining. The adaptation strategy used in this study does not implement explicit domain-adversarial or discrepancy-minimization objectives. Instead, it represents an intermediate retinal-domain fine-tuning stage intended to improve feature alignment prior to downstream diabetic retinopathy classification.

### 3.5 Channel-spatial attention module (CSAM) ablation

In selected configurations, a Channel–Spatial Attention Module (CSAM) was optionally inserted before the final classification head. CSAM reweights feature maps along both channel and spatial dimensions so that potentially informative regions receive greater emphasis while less relevant responses are reduced. Channel attention focuses on discriminative feature channels, and spatial attention highlights informative retinal areas. Since transformer backbones already include internal attention mechanisms and fusion models combine heterogeneous feature representations, it was not assumed that CSAM would always be beneficial. Instead, its effect was evaluated through systematic ablation across standalone and fusion setups, and its contribution was interpreted based on measured class-balanced metrics rather than architectural expectation.

### 3.6 Backbone architectures

#### 3.6.1 ConvNeXt-Tiny

ConvNeXt-Tiny (Liu et al., 2022) was selected as the CNN backbone in this study because it modernizes conventional convolutional network design while preserving convolutional inductive biases that are beneficial for localized retinal lesion analysis. The architecture incorporates large-kernel depthwise convolutions, hierarchical feature extraction, and transformer-inspired design refinements while maintaining the efficiency of convolutional processing.

In retinal fundus imaging, fine-grained pathological patterns such as microaneurysms, hemorrhages, and vessel irregularities often require strong local feature sensitivity. ConvNeXt-Tiny is therefore well-suited for extracting lesion-level representations relevant to diabetic retinopathy grading. The architecture follows a four-stage hierarchical design with progressive downsampling and increasing channel dimensions, enabling the model to learn both low-level retinal textures and higher-level semantic representations.

In this study, ConvNeXt-Tiny was initialized with ImageNet-1K pretrained weights and evaluated under both direct fine-tuning and domain-adaptive transfer learning settings. Feature representations extracted from the final stage were used either for standalone classification or for feature fusion with Swin-Tiny within the proposed hybrid framework.

#### 3.6.2 Swin-Tiny

Swin-Tiny (Liu et al., 2021) was selected as the transformer-based backbone in this study because it provides hierarchical contextual modeling through shifted-window self-attention while maintaining relatively low computational complexity compared with standard Vision Transformers. Unlike convolutional architectures that primarily emphasize localized receptive fields, Swin-Tiny can capture broader spatial dependencies and contextual relationships across retinal regions.

In retinal fundus imaging, diabetic retinopathy severity is influenced not only by isolated lesions but also by their spatial distribution and contextual relationships within the retina. The hierarchical shifted-window attention mechanism in Swin-Tiny therefore provides an effective framework for modeling both local retinal structures and wider contextual information relevant to disease grading.

The architecture follows a multi-stage hierarchical design with progressive patch merging and feature abstraction. In this study, Swin-Tiny was initialized with ImageNet-1K pretrained weights and evaluated under both direct fine-tuning and domain-adaptive transfer learning settings. Features extracted from the final stage were used either for standalone diabetic retinopathy classification or for feature fusion with ConvNeXt-Tiny within the proposed hybrid framework.

### 3.7 Proposed hybrid fusion model (ConvNeXt-Tiny+Swin-Tiny)

**Figure 4:**
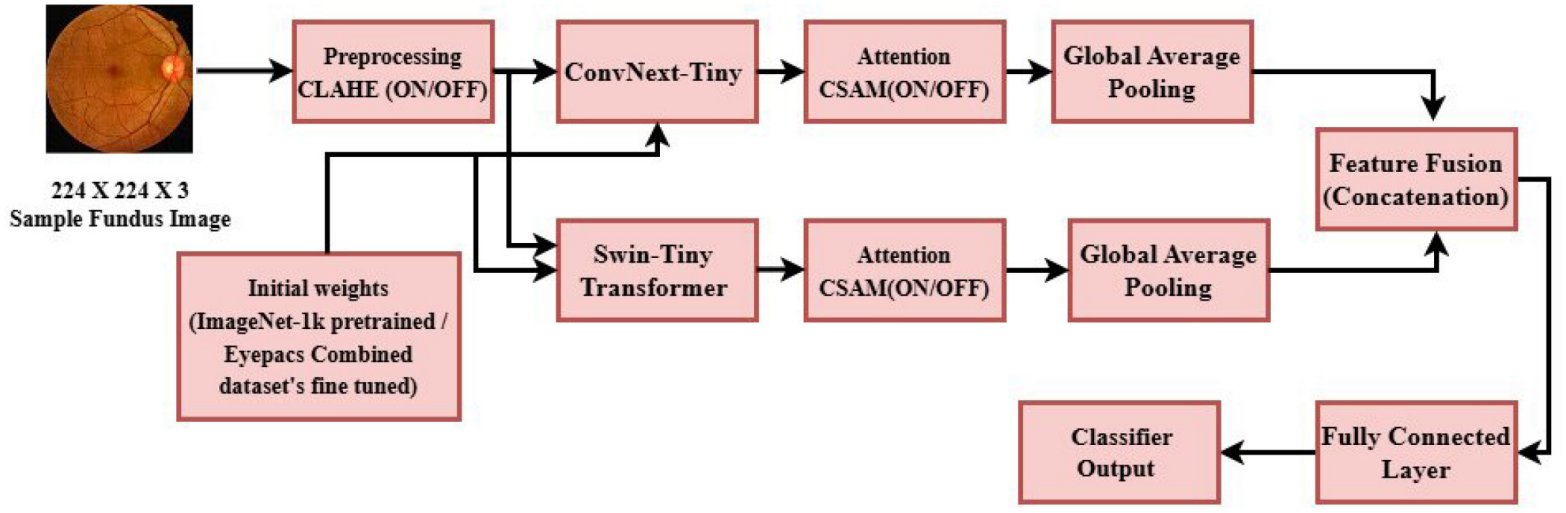
Proposed hybrid fusion model.

The proposed hybrid fusion model, illustrated in Figure 4, combines ConvNeXt-Tiny and Swin-Tiny to capture both fine-scale retinal details and broader contextual spatial dependencies. The fusion framework enables complementary representation learning by integrating convolutional lesion-sensitive features with transformer-based contextual representations. While the ConvNeXt-Tiny and Swin-Tiny architectures share a remarkably similar hierarchical design, they are fundamentally different in how they extract and process features. ConvNeXt-Tiny creates its feature hierarchy through convolutional operations with large receptive fields that enable effective spatial relationship modeling, while progressively learning increasingly complex feature representations with network depth. In contrast, Swin-Tiny creates hierarchical representations using windowed self attention, which computes attention in local windows and then shifts the location of the window across layers to enable cross-window interactions. Despite their different feature extraction mechanisms, both models share a four-stage hierarchical pyramid structure that produces feature maps with compatible spatial resolutions and channel dimensions. This architectural alignment of each stage enables feature-level fusion compatibility of ConvNeXt-Tiny and Swin-Tiny, thus facilitating their hybrid integration in visual understanding tasks such as diabetic retinopathy classification.

#### 3.7.1 Features fusion

Feature maps are extracted from ConvNeXt-Tiny and Swin-Tiny at the output of their final stages. In this implementation, ConvNeXt-Tiny contributed fine-grained local descriptors, while Swin-Tiny provided features that encode broader contextual structure. Within the ablation framework, the CSAM attention module was optionally applied to each branch so that its contribution could be evaluated directly instead of being treated as inherently beneficial. This setup enables evaluation of the practical impact of attention-based feature reweighting across different configurations. Global average pooling was then applied to the output of each branch to produce compact global descriptors with matched dimensionality. The pooled feature vectors from the two backbones are concatenated along the channel dimension to build a fused representation that combines lesion sensitive local information with global contextual cues. Using global average pooling keeps the fusion stage lightweight, reduces spatial redundancy, and maintains stable feature compatibility between branches. The resulting fused descriptor is passed to the classification head for final prediction. By combining ConvNeXt-Tiny local detail sensitivity with Swin-Tiny global dependency modeling in this pooled feature space, the fusion design supports complementary representation learning, while the ablation experiments verify whether the added attention mechanism provides a measurable benefit.

#### 3.7.2 Inference strategy

At inference, Test-Time Augmentation (TTA) was applied using six augmented image views: identity, horizontal flip, rotations of +10° and −10°, central crop, and scaled crop. Predictions from all augmented views were averaged to obtain the final output. This strategy improved robustness against variations in retinal orientation, illumination, and image quality.

#### 3.7.3 Hardware configuration

All experiments were conducted using the Kaggle cloud computing environment equipped with dual NVIDIA Tesla T4 GPUs (*T*4 × 2). Each GPU provides 16 GB of GDDR6 memory and is based on the Turing architecture, offering efficient support for mixed-precision training and large-scale deep learning workloads. The compute environment was further supported by Intel Xeon CPUs, approximately 30 GB of system RAM, and high-speed SSD storage, ensuring efficient data loading and parallel processing during training and evaluation.

## 4 Results

### 4.1 Result analysis

Tables 2–4 summarize the comparative performance of fusion and standalone architectures under different combinations of domain adaptation, CLAHE preprocessing, and CSAM integration. Test accuracy, precision, recall, and macro F1-score were evaluated for all configurations. To assess reproducibility and training stability, all experiments were repeated across three random seeds, and performance metrics are reported as mean ± standard deviation values.

**Table 2:** Results for ConvNeXt-Tiny and Swin-Tiny fusion models.

Model no	Domain adaptation	CLAHE & CSAM	Test acc	Test prec	Test recall	Test macro F1-score
2.1	Yes	Yes	84.15 ± 0.27	0.7513 ± 0.1335	0.5536 ± 0.0059	0.5586 ± 0.0086
2.2	Yes	No	88.34 ± 1.09	0.8063 ± 0.0358	0.7063 ± 0.0154	0.7376 ± 0.0183
2.3	No	Yes	80.60 ± 1.09	0.6825 ± 0.0669	0.5128 ± 0.0149	0.5295 ± 0.0222
2.4	No	No	86.70 ± 1.03	0.7626 ± 0.0387	0.6831 ± 0.0151	0.7096 ± 0.0209

**Table 3:** Results for ConvNeXt-Tiny models.

Model no	Domain adaptation	CLAHE & CSAM	Test acc	Test prec	Test recall	Test macro F1-score
3.1	Yes	Yes	75.68 ± 3.43	0.5601 ± 0.0732	0.4682 ± 0.0463	0.4656 ± 0.0574
3.2	Yes	No	87.52 ± 0.82	0.8042 ± 0.0162	0.6915 ± 0.0150	0.7267 ± 0.0177
3.3	No	Yes	80.97 ± 1.10	0.6825 ± 0.0664	0.5128 ± 0.0415	0.5818 ± 0.0564
3.4	No	No	83.97 ± 1.56	0.7211 ± 0.0101	0.6261 ± 0.0298	0.6496 ± 0.0329

**Table 4:** Results for Swin-Tiny models.

Model no	Domain adaptation	CLAHE & CSAM	Test Acc	Test Prec	Test recall	Test macro F1-Score
4.1	Yes	Yes	54.37 ± 0.00	0.1087 ± 0.0000	0.2000 ± 0.0000	0.1409 ± 0.0000
4.2	Yes	No	54.37 ± 0.00	0.1087 ± 0.0000	0.2000 ± 0.0000	0.1409 ± 0.0000
4.3	No	Yes	82.79 ± 1.66	0.7135 ± 0.0932	0.5551 ± 0.0408	0.5818 ± 0.0559
4.4	No	No	86.07 ± 0.73	0.7456 ± 0.0109	0.6517 ± 0.0249	0.6785 ± 0.0253

**Figure 5:**
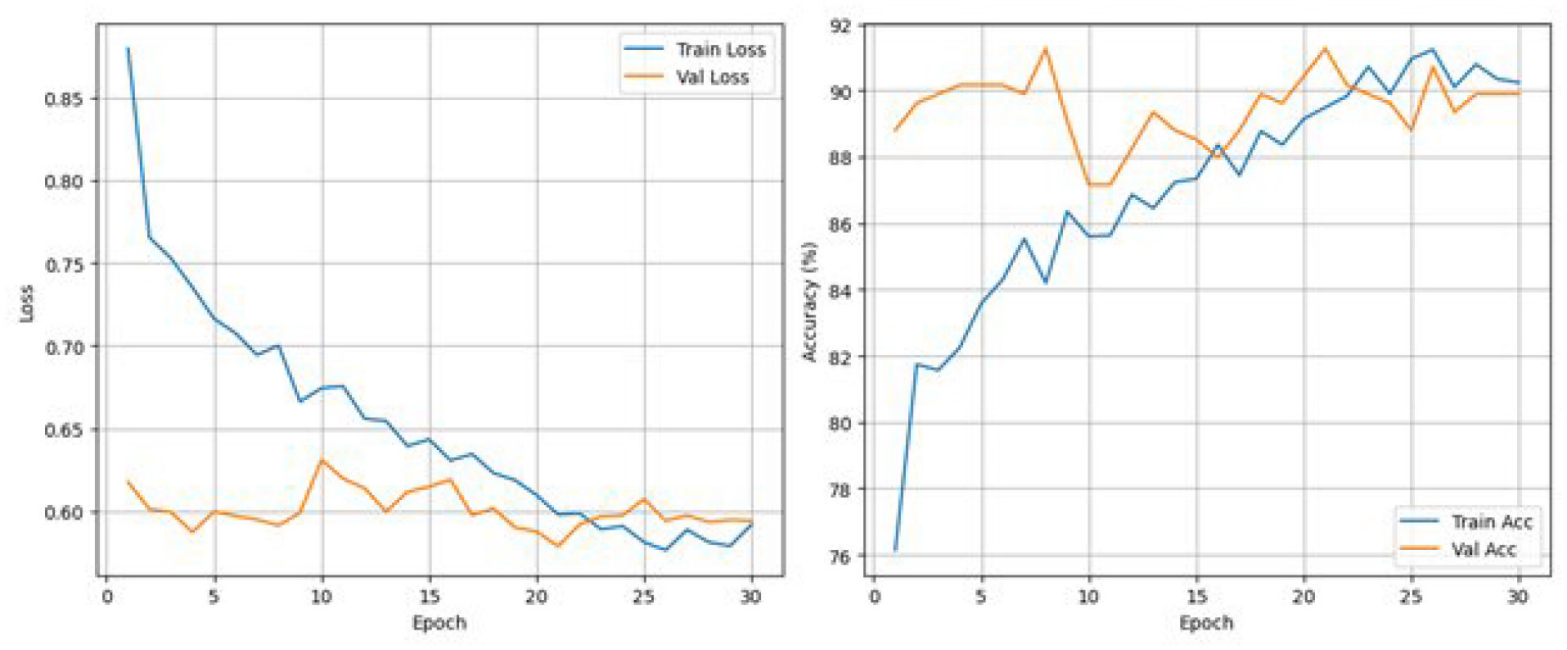
Loss and accuracy curves of fusion model 2.2.

**Figure 6:**
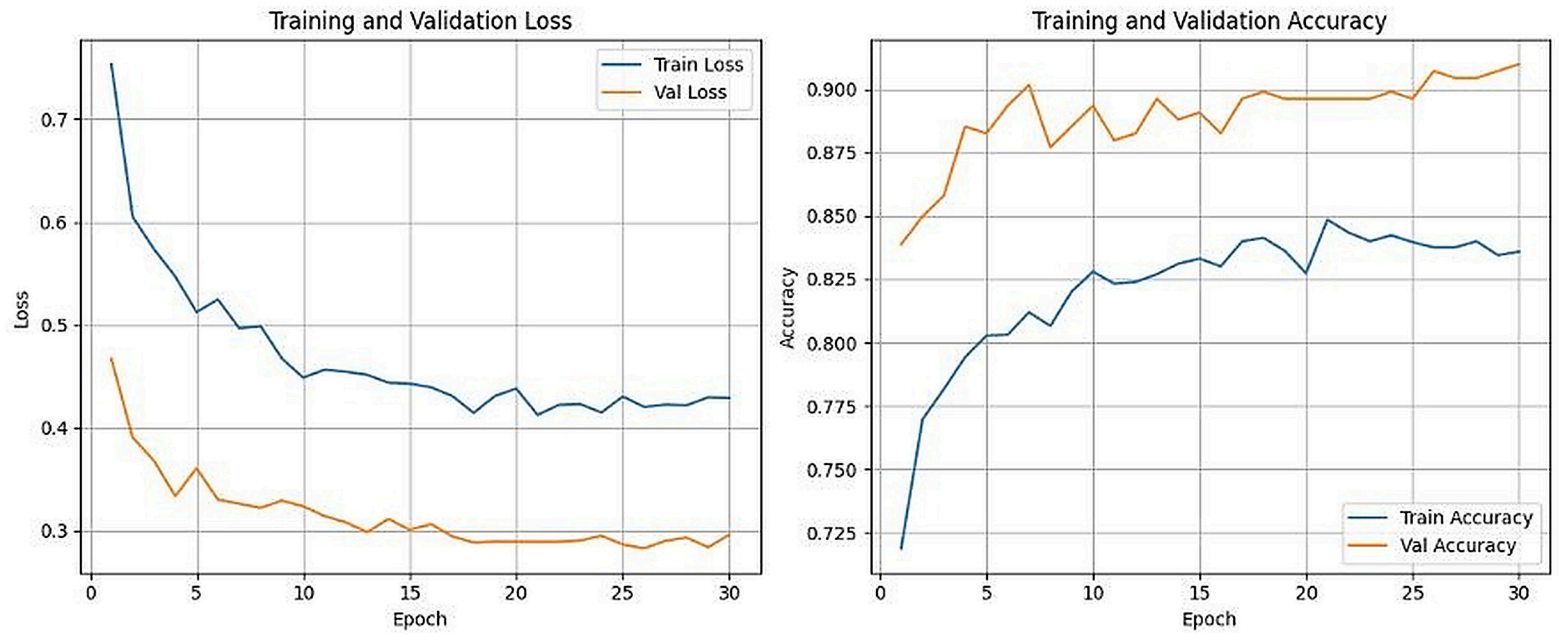
Loss and accuracy curves of ConvNeXt-Tiny model (3.2).

**Figure 7:**
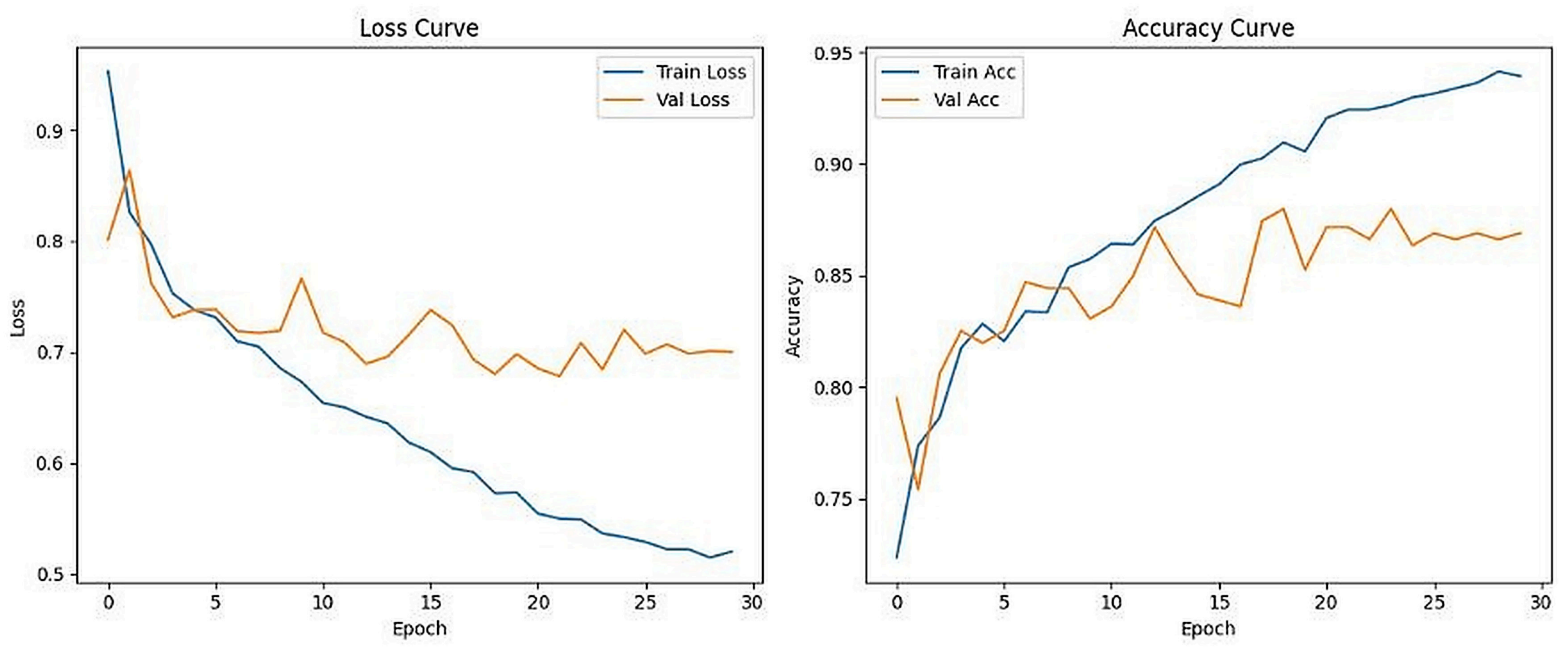
Loss and accuracy curves of Swin-Tiny model (4.4).

Figures 5–7 present representative training and validation loss and accuracy curves for selected configurations: (i) fusion with EyePACS-based domain adaptation without CLAHE and CSAM, (ii) ConvNeXt-Tiny with domain adaptation without CLAHE and CSAM, and (iii) Swin-Tiny with ImageNet-1K initialization without CLAHE and CSAM. Across experiments, convergence behavior varied with both architecture and configuration.

The curves in Figure 5 show comparatively smooth and stable convergence, with steadily decreasing training and validation loss and closely aligned accuracy trends, indicating stable optimization and effective generalization. In Figure 6, validation accuracy exceeds training accuracy during several epochs, a behavior consistent with regularization effects introduced by augmentation and stochastic optimization. Figure 7 shows mild separation between training and validation curves during later epochs, suggesting moderate overfitting behavior. Among these representative runs, the fusion configuration in Figure 5 exhibited the most stable convergence pattern.

**Figure 8:**
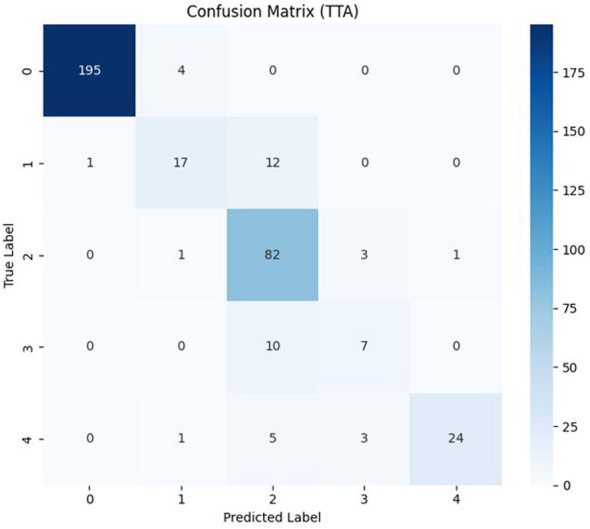
Confusion matrix of fusion model (2.2).

**Figure 9:**
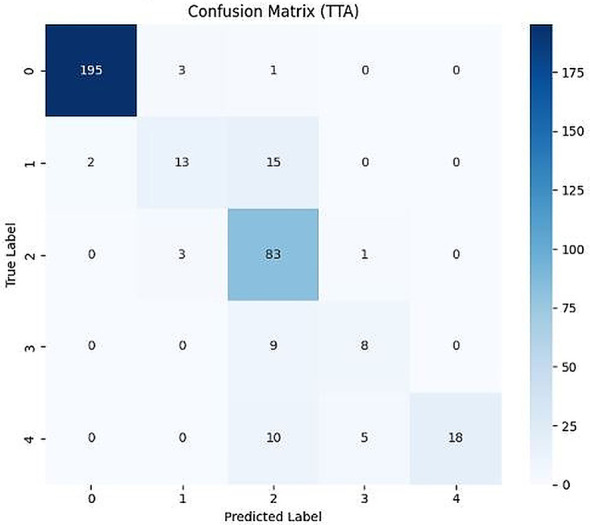
Confusion matrix of ConvNeXt-Tiny model (3.2).

**Figure 10:**
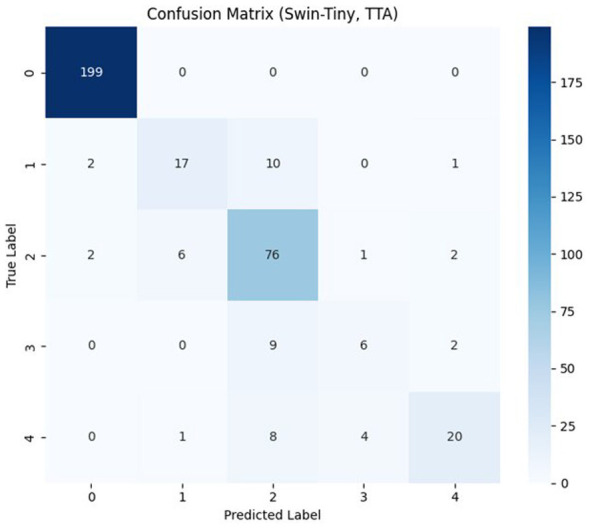
Confusion matrix of Swin-Tiny model (4.4).

Class-wise behavior was further analyzed using the confusion matrices shown in Figures 8–10. The confusion matrix corresponding to the fusion configuration (Figure 8) demonstrates strong recognition of majority and intermediate DR classes, with most misclassifications occurring between clinically adjacent grades such as Mild and Moderate DR. Figure 9 shows comparatively higher confusion between neighboring classes in the standalone ConvNeXt-Tiny configuration. Figure 10 demonstrates relatively balanced recognition performance for Swin-Tiny in its best-performing configuration, although some confusion persists between Moderate, Severe, and Proliferative DR stages. Overall, the confusion matrices suggest that most classification errors arise from inter-class visual similarity rather than severe systematic bias.

#### 4.1.1 Fusion models results

Table 2 summarizes the fusion-based configurations evaluated under different combinations of domain adaptation, CLAHE preprocessing, and CSAM integration. Fusion performance was strongly dependent on training configuration and was not uniformly superior across all settings. Among the evaluated fusion variants, configuration 2.2, which combines EyePACS-based domain adaptation without CLAHE and CSAM, achieved the strongest average performance with a mean test accuracy of 88.34%±1.09 and a macro F1-score of 0.7376 ± 0.0183.

Fusion configurations incorporating CLAHE and CSAM generally produced LOWER recall and macro F1-score values, indicating reduced class-balanced classification performance under these settings. These observations suggest that additional contrast enhancement and external attention mechanisms do not consistently improve class-balanced diabetic retinopathy grading performance. Fusion models initialized only from ImageNet-1K weights (Models 2.3 and 2.4) demonstrated weaker average performance than the corresponding domain-adapted fusion configurations, indicating that retinal domain alignment contributed positively to fusion behavior under the evaluated conditions.

**Figure 11:**
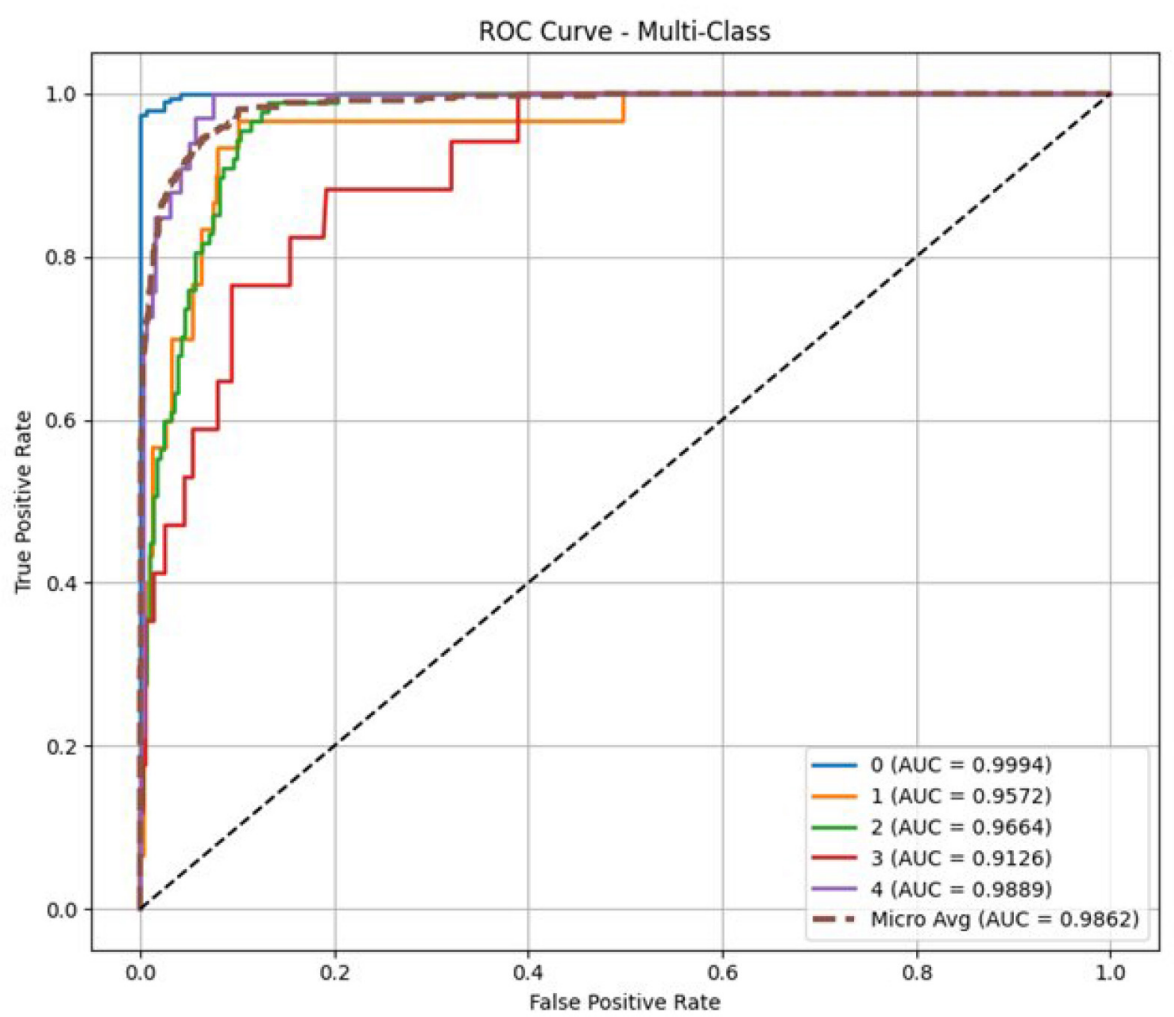
ROC curve of proposed model (2.2).

To further evaluate the discriminative capability of the best-performing fusion configuration, multiclass ROC analysis was performed and the corresponding ROC curves are presented in Figure 11, while the quantitative AUC values are summarized in Table 5. The model achieved a micro-average ROC-AUC score of 0.9862, indicating strong overall separability across diabetic retinopathy severity classes. Class-wise AUC values were also consistently high, with the strongest discrimination observed for Class 0 (AUC = 0.9994) and Class 4 (AUC = 0.9889). Moderate performance reduction was observed for Class 3 (AUC = 0.9126), which is likely related to increased visual similarity and overlap between adjacent severe retinal disease stages. Overall, the ROC-AUC analysis further supports the robustness of the proposed fusion framework and demonstrates effective class discrimination across both majority and minority DR categories.

**Table 5:** AUC results for the best-performing fusion configuration.

Class	AUC value	Micro average AUC
Class 0	0.9994	0.9862
Class 1	0.9572	
Class 2	0.9664	
Class 3	0.9126	
Class 4	0.9889	

#### 4.1.2 Standalone ConvNeXt-Tiny models results

Table 3 presents the standalone ConvNeXt-Tiny configurations evaluated under the same combinations of domain adaptation, CLAHE preprocessing, and CSAM integration. The domain-adapted ConvNeXt-Tiny configuration without CLAHE and CSAM (Model 3.2) delivered the strongest standalone CNN performance, with a mean test accuracy of 87.52%±0.82 and a macro F1-score of 0.7267 ± 0.0177 across repeated runs.

Compared with the corresponding ImageNet-1K initialized configurations, the domain-adapted ConvNeXt-Tiny models demonstrated improved and comparatively stable performance, supporting the usefulness of retinal domain alignment for convolutional backbones. Configurations incorporating CLAHE and CSAM showed consistent reductions in recall and macro F1-score. In this study, additional contrast enhancement and attention-based reweighting were observed to reduce sensitivity to subtle lesion-related representations when combined with an already well-regularized convolutional architecture.

#### 4.1.3 Standalone Swin-Tiny model results

Table 4 summarizes the standalone Swin-Tiny configurations. Compared with ConvNeXt-Tiny, Swin-Tiny demonstrated substantially greater sensitivity to the domain adaptation stage. The EyePACS-adapted Swin-Tiny configurations (Models 4.1 and 4.2) converged to substantially weaker performance, with mean test accuracy around 54.37% and macro F1-score near 0.14. The near-identical behavior across repeated runs suggests unstable optimization behavior under this adaptation setting.

By contrast, Swin-Tiny configurations initialized from ImageNet-1K and directly fine-tuned on APTOS 2019 achieved substantially stronger results. Model 4.4 achieved a mean test accuracy of 86.07%±0.73 and a macro F1-score of 0.6785 ± 0.0253. Although competitive, the standalone Swin-Tiny configurations remained below ConvNeXt-Tiny and the fusion models in recall and macro F1-score, indicating that global attention mechanisms alone were comparatively less effective than convolutional inductive bias for subtle lesion-sensitive retinal classification under the evaluated conditions.

### 4.2 Comparative result discussion

Across the evaluated configurations, performance patterns were found to depend strongly on both architecture selection and transfer learning strategy. The fusion configuration with EyePACS-based domain adaptation and without CLAHE or CSAM (Model 2.2) achieved the strongest overall average performance, with a mean test accuracy of 88.34%±1.09 and a macro F1-score of 0.7376 ± 0.0183. These results suggest that combining convolutional and transformer-based representations can improve class-balanced diabetic retinopathy grading when domain alignment is appropriately matched to the architecture. At the same time, fusion did not improve all evaluation metrics uniformly. Improvements in minority-class recall remained moderate, indicating that increased architectural complexity alone does not guarantee proportional gains in class-balanced performance.

The effect of EyePACS-based domain adaptation was found to be highly architecture-dependent in this study. Domain adaptation consistently improved ConvNeXt-Tiny and fusion configurations, whereas it substantially reduced performance for Swin-Tiny. This contrast indicates that domain adaptation cannot be assumed universally beneficial across backbone families and should instead be evaluated separately for different architectural paradigms. CLAHE preprocessing and CSAM integration were also observed to reduce macro recall and macro F1-score across several configurations. Although CLAHE improves local contrast visually, the resulting enhancement did not consistently translate into improved class-balanced classification performance and in some cases appeared to reduce sensitivity to subtle lesion-related features. Similarly, adding external attention modules on top of already attention-capable or strongly regularized backbones did not provide reliable performance gains under the evaluated settings.

To provide contextual comparison, Table 6 summarizes representative studies evaluated on the APTOS 2019 dataset. Kobat and Yildirim (2022) proposed a DenseNet–SVM hybrid framework with patch-based feature extraction and reported a validation accuracy of 87.43%. Ahmed (2025) implemented an EfficientNet-based transfer learning framework for class imbalance handling and achieved a test accuracy of 84.6%. Mohsen et al. (2025) introduced the RadFuse framework using RadEx-transformed retinal representations with a ResNeXt-50 backbone and reported a test accuracy of 87.07%. In comparison, the proposed fusion configuration achieved a best single-run test accuracy of 89.34% and a mean test accuracy of 88.34% ± 1.09 across repeated runs. However, because prior studies used different dataset partitions, preprocessing pipelines, augmentation strategies, and evaluation protocols, these comparisons should be interpreted cautiously rather than as direct state-of-the-art rankings.

**Table 6:** Performance comparison of the proposed fusion model with prior methods.

Reference/ model	Dataset	Method	Test acc
Kobat and Yildirim (2022)	APTOS 2019	DenseNet-SVM	—
Ahmed (2025)	APTOS 2019	EfficientNet	0.8460
Mohsen et al. (2025)	APTOS 2019	RadFuse (ResNeXt)	0.8707
Proposed fusion model (2.2)	APTOS 2019	(ConvNeXt-Tiny + Swin-Tiny) fusion	88.34 ± 1.09

From these controlled comparisons, three major observations emerge. First, convolutional backbones continue to provide strong lesion-sensitive representations for diabetic retinopathy grading. Second, hybrid fusion can improve balanced classification performance when domain adaptation is appropriately aligned with the underlying architecture. Third, auxiliary preprocessing and attention mechanisms are best justified through systematic ablation evidence rather than assumed benefit.

## 5 Conclusion

In this study, we present a controlled comparison of ConvNeXt-Tiny, Swin-Tiny and variants of both networks either used as stand-alone networks or in a feature fusion configuration for diabetic retinopathy classification using retinal fundus images. All models were initialized with ImageNet-1K pretrained weights and evaluated under two transfer learning strategies, direct fine-tuning and EyePACS-based domain adaptation. Moreover, systematic ablation experiments with CLAHE preprocessing and channel-spatial attention modules (CSAM) were performed to explore their respective contributions under the same experimental settings. All configurations were run three times with different random seeds to check reproducibility and training stability and the results were reported as mean ± standard deviation.

Model performance varied significantly across experiments depending on backbone architecture and transfer learning configuration. ConvNeXt-Tiny presented stable and competitive performance, always benefitting from domain adaptation, achieving a mean test accuracy of 87.52%±0.82 and a macro F1-score of 0.7267 ± 0.0177 in its best configuration. In contrast, domain-adapted Swin-Tiny showed significantly lower and more unstable performance under class imbalance conditions, indicating that transformer-based retinal representations are still sensitive to dataset characteristics and adaptation strategy. We found that Swin-Tiny performed better when fine-tuning directly from ImageNet-1K initialization. Its best single setup achieved a mean test accuracy of 86.07%±0.73 and a macro F1-score of 0.6785 ± 0.0253.

The best overall average performance among all configurations evaluated was achieved by the fusion-based framework with domain adaptation based on EyePACS and without CLAHE or CSAM, with a mean test accuracy of 88.34%±1.09 and a macro F1-score of 0.7376 ± 0.0183. These results indicate that combining convolutional and transformer-based feature representations can improve class-balanced diabetic retinopathy grading when domain alignment is properly matched with the architecture. The ablation experiments also revealed that CLAHE preprocessing and CSAM integration were not consistently improving macro recall or macro F1-score and even degrading class-balanced performance in some configurations. Collectively, our results emphasize the importance of backbone selection, transfer learning strategy, and meticulous fusion design over auxiliary enhancement techniques in diabetic retinopathy classification workflows.

There are also a few limitations of the present study to be acknowledged. First, the evaluation was primarily conducted on the APTOS 2019 dataset without external validation on independent retinal imaging datasets, which limits conclusions regarding broader clinical generalizability. Second, while the fusion framework improved the overall balance on performance metrics, it also introduced increased computational complexity and training overhead compared to standalone lightweight architectures. Finally, Patient-level separation and rigorous duplicate-image verification were not independently verifiable because of the absence of patient metadata in the processed public dataset structure.

Future work will involve external multi-dataset validation, exploration of computationally-efficient fusion strategies, and incorporation of Explainable AI (XAI) methods to improve interpretability and clinical transparency of model decision-making behavior. In conclusion, the results support the value of controlled comparative evaluation for understanding the practical behavior of CNN-based, transformer-based and hybrid architectures for diabetic retinopathy grading.

## Data Availability

The original contributions presented in the study are included in the article/supplementary material, further inquiries can be directed to the corresponding author.
